# The effect of health literacy on quality of life in patients with heart failure: the mediating role of self-care confidence

**DOI:** 10.3389/fcvm.2026.1772842

**Published:** 2026-07-01

**Authors:** Qian Yao, Meng Cao, Yu Ni, Ting Xu, Chunxia He, Xiuchuan Li, Jimei Zhang

**Affiliations:** 1 Emergency Department, The Third People’s Hospital of Chengdu, Chengdu, China; 2 School of Nursing, Chengdu Medical College, Chengdu, China; 3 School of Nursing, Sichuan Vocational College of Health and Rehabilitation, Zigong, China; 4 College of Nursing, Chengdu University of Traditional Chinese Medicine, Chengdu, China; 5 Department of Cardiology, The General Hospital of Western Theater Command PLA, Chengdu, China

**Keywords:** health literacy, heart failure, mediating role, quality of life, self-care confidence

## Abstract

**Background:**

Heart failure (HF) is a global epidemic that seriously affects the quality of life (QoL) of most patients. Self-care is an important factor affecting QoL, and health literacy (HL) plays a key role in self-care. However, the relationship between HL, self-care and QoL in patients with HF remains unclear.

**Objectives:**

To explore whether self-care confidence mediates the relationship between HL and QoL in patients with HF.

**Methods:**

A total of 320 HF patients from three hospitals in Chengdu, China were selected as the research subjects. Data were collected using the Chinese version of the HF HL scale, self-care confidence scale, and QoL scale. The influencing factors of QoL were analyzed by independent samples t-test, one-way analysis of variance, and multiple linear regression. Pearson correlation analysis was used to analyze the correlation between HL, self-care confidence and QoL. Mediating effects were analyzed and validated using the PROCESS macro (Model 4) and Bootstrap method.

**Results:**

The HL score was (24.45 ± 9.44) points, the self-care confidence score was (33.66 ± 18.63) points, and the QoL score was (48.50 ± 13.32) points. HL was positively correlated with self-care confidence (*r* = 0.807, *P* < 0.01), and negatively correlated with QoL (*r* = −0.472, *P* < 0.01). Self-care confidence was negatively correlated with QoL (*r* = −0.454, *P* < 0.01). The total effect value of HL on QoL was −0.666, and the mediating effect value was −0.240. Self-care confidence played a mediating role between HL and QoL, and the mediating effect accounted for 36%.

**Conclusions:**

HL significantly impacts QoL, and self-care confidence mediates the relationship between HL and QoL. Therefore, in the care of HF patients, healthcare professionals should focus on improving patients' HL and self-care confidence. It is recommended to implement targeted health education programs for patients in clinical practice, emphasizing disease management knowledge and cultivating self-care confidence to further reduce the risk of readmission for HF patients and optimize overall prognosis.

## 1 Introduction

Heart failure (HF) is a complex clinical syndrome caused by abnormal cardiac structure and/or function that impairs ventricular filling and/or ejection function. It is usually accompanied by objective signs such as elevated natriuretic peptide levels and pulmonary or systemic congestion. It is a late stage of heart disease (1). The prevalence of HF in people aged 65–79 and ≥80 years in China is 3.86% and 7.55%, respectively (2). Therefore, the prevalence of HF in the elderly population in China is extremely high, and the prevalence tends to increase gradually with age. In fact, HF is a globally prevalent disease. Currently, approximately 64.3 million people suffer from HF worldwide, and HF is also the leading cause of morbidity and mortality globally (3, 4). HF has become a major public health concern globally, and its treatment and management have drawn widespread attention from countries around the world.

With the advancement of medical science, the focus of modern HF management has shifted from prolonging patients' life to alleviating symptoms, improving patients' functional ability and improving patients' quality of life (QoL) (4, 5). QoL refers to patients' subjective feelings about their life goals, expectations, standards and things they care about in different cultural and value contexts (6). Since QoL can evaluate patients' physical, psychological and family social conditions and more comprehensively reflect patients' health status in the recent period, it has become the main prognostic indicator for HF research. Studies have shown that (7) a decrease in QoL will not only lead to an increase in the hospitalization rate of HF patients, but also an increase in mortality. When the QoL score of HF patients increases by 10 points, their all-cause mortality risk will increase by 12% (95% CI = 6%–18%) (8). Kraai et al. found that (9) 61% of patients were more concerned about improving QoL rather than the length of life, and even 14% of patients were willing to exchange 12 months of life for a higher level of QoL. This further emphasizes the importance of improving QoL in HF management. A systematic review found that (10) the average QoL score of HF patients was 44.1 points (95% CI = 40.6–47.5), indicating that the QoL of most patients was at a moderate or low level. This finding highlights the urgency of improving the QoL of HF patients and suggests that we need to take effective intervention measures to improve patients' daily life experiences (5).

In order to improve the QoL of HF patients, we need to understand the factors that affect QoL. Self-care refers to the process of maintaining health through health promotion and prevention practices, including restricting fluid and salt intake, taking medications consistently, identifying symptoms and taking timely treatment measures (11). Self-care is crucial for HF patients and is the focus of multidisciplinary HF management programs worldwide. The situation-specific theory of HF self-care proposed by Riegel et al. emphasizes that good self-care behaviors can improve the health status of HF patients, and that self-care skills above a moderate level are required to effectively improve the health status of patients (12). In addition, another theory of Riegel et al., the chronic disease self-care theory, further points out that the results of chronic disease self-care include QoL, disease stability, happiness, perceived control, health care costs and mortality, but the results of self-care are mainly reflected in QoL (13). Related studies have also shown that self-care correlates with QoL, and patients with good self-care behaviors usually have better QoL and lower mortality and readmission rates (6, 11). Therefore, we can infer that self-care is an important factor affecting QoL.

Health literacy (HL) refers to an individual's ability to obtain and understand health information and use this information to maintain and promote their own health (14). The situation-specific theory of HF self-care proposes that self-care is influenced by the interaction of person (knowledge, skills, self-efficacy, HL), problem (cardiac functioning classification, instrumental activities of daily living), and environmental (social support, living areas) factors (15, 16). Therefore, we can know that HL is one of the influencing factors of self-care. In addition, previous studies have shown that (17–20) HL is significantly associated with self-care behaviors, and that increasing the level of HL among HF patients can promote improved self-care behaviors. This further confirms that HL may be an important factor influencing patients' self-care behaviors.

The situation-specific theory of HF self-care states that self-care is a natural decision-making process consisting of self-care maintenance (behaviors taken to maintain physiological stability), symptom perception (the ability to monitor and recognize symptoms), and self-care management (The ability to handle symptoms as they occur) (21). This process includes a very important concept, namely self-care confidence, which refers to the patient's subjective assessment of whether he or she can complete self-care activities when facing difficulties and challenges (12). Self-care confidence itself is not part of the self-care process, but it is an extremely important factor that affects the effectiveness of self-care behavior. Self-care confidence plays a key role in promoting the transformation of knowledge into actual self-care behavior (22). Therefore, it is included in the self-care model (19, 23, 24). The theory also points out that self-care confidence has a mediating role between predictor variables and outcome variables (12, 21). As shown in previous studies (19, 24–27), self-care confidence plays an important mediating role between multiple variables (such as HL, knowledge, social support, etc.) and outcome variables.

Based on previous research, we know that self-care is a crucial factor influencing QoL, and self-care is further influenced by HL levels. Therefore, we have reason to speculate that self-care may mediate the relationship between HL and QoL. Furthermore, combining this with the context-specific theory of self-care in HF, we know that self-care confidence is a significant factor influencing self-care, and it has repeatedly played a mediating role in studies. Therefore, this study proposes the following hypotheses: 1. HL significantly affects the QoL of HF patients; 2. Self-care confidence mediates the relationship between HL and QoL in HF patients. This study not only explores the key factors influencing the QoL of HF patients but also aims to reveal the pathway of influence: “HL → self-care confidence → QoL.” Through this study, we hope to identify key interventionable links, providing empirical evidence for developing targeted health education strategies and self-care confidence enhancement programs in clinical practice, ultimately optimizing disease management and QoL for HF patients.

## 2 Methods

### 2.1 Study design and participants

A cross-sectional questionnaire survey was conducted in three tertiary hospitals in Chengdu, Sichuan Province, China from October 2022 to July 2023. Convenience sampling was used to recruit HF patients. The inclusion criteria were as follows: (1) patients who met the diagnostic criteria for HF and were diagnosed with HF by a cardiologist; (2) New York Heart Association (NYHA) functional class II-IV; (3) age ≥18 years; (4) voluntarily participating in this study and signing an informed consent form. The exclusion criteria were as follows: (1) class IV patients suffering from multiple organ failure; (2) patients with psychiatric and cognitive disorders who could not understand the questionnaire content and could not communicate; (3) patients after heart transplantation; (4) patients with malignant tumors.

This study used the cross-sectional sample size calculation formula $n=\frac{Z_{1-a/2}^{2}\sigma^{2}}{d^{2}}$ (28), setting *α* as 0.05, then Z_(1−*α*/2)_ was 1.96. The standard deviation of QoL scores was 18.63 through preliminary survey analysis, and the precision d was set to 2.3, resulting in *n* = 253. Considering the 20% dropout rate, the minimum sample size was determined to be 304 cases. In this study, 330 questionnaires were actually distributed. During the data review process, we excluded those questionnaires with missing items or obvious regular deviations in the answers, a total of 10 questionnaires. Finally, 320 valid questionnaires were obtained, and the questionnaire recovery rate reached 96.97%.

### 2.2 Data collection

Before the formal investigation, 30 patients (not included in the analysis) were pre-surveyed. Each survey required the patient to be introduced to the purpose, precautions and survey methods of this study, and to ensure the anonymity and after obtaining the patient's consent and signing the informed consent form. Those who have the ability to write can fill in the questionnaire by themselves. For patients with low cultural levels and illiteracy, investigators confidentiality of the questionnaire. The survey can only be conducted can assist patients to complete the questionnaire by asking and explaining, but they should be careful not to guide patients to answer implicitly. When the patient fills in the questionnaire, it is collected immediately, and the questionnaire is checked for omissions, random answers, etc. For problematic questionnaires, the patient should be asked the reason in time and supplemented accordingly.

The design of this study strictly followed the principles of the Declaration of Helsinki and was approved by the Ethics Review Committee of Chengdu Medical College (2022NO.48) before data collection.

### 2.3 Measures

#### 2.3.1 Sociodemographic and clinical characteristics

The sociodemographic and clinical characteristics of the participants were designed by our research members after reviewing relevant literature. The questionnaire included gender, age, marital status, living area, education level, work status, average monthly income, left ventricular ejection fraction (LVEF), New York Heart Association (NYHA) functional class, comorbidities, and HF duration.

#### 2.3.2 Health literacy

The Heart Failure-Specific Health Literacy Scale (HF-specific HL scale) developed by Matsuoka was used to measure HL (29). The HF-specific HL scale consists of 3 dimensions (functional HL, communicative HL, and critical HL) and 12 items. A Likert 1-4 scale was used, with a total score of 12-48, with higher scores representing higher levels of HL. The Chinese version of the HF-specific HL scale was used for measurement (30), which has a Cronbach's alpha coefficient of 0.870, and in this study the Cronbach's alpha coefficient was 0.962.

#### 2.3.3 Self-care confidence

Riegel developed the Self-Care of Heart Failure Index (SCHFI) in 2004 to measure the self-care ability of HF patients (31). In 2009, Riegel revised the scale to form the SCHFIv6.2 version (23). SCHFIv6.2 contains three subscales, self-care maintenance (10 items), self-care management (6 items), and self-care confidence (6 items), and all three subscales can be used individually. In this study, the self-care confidence subscale from SCHFIv6.2 was used for measurement. Each item was scored using the Likert 1–4 scale, and the total score was calculated using the conversion score. The scoring method was = (original score-minimum score)/(maximum score-minimum score)  ×   100, and the specific calculation method was = (original score-6)/(24−6) × 100. The total score was 100, and a score of higher scores represent stronger self-care confidence, and >70 points indicate better self-care confidence. In this study, the Chinese version of the Self-care confidence scale was used for measurement (32), which has a Cronbach's alpha coefficient of 0.870, and the Cronbach's alpha coefficient was 0.849 in this study.

#### 2.3.4 Quality of life

The Minnesota Living with Heart Failure Questionnaire (MLHFQ) was developed by Rector et al. to investigate the QoL of HF patients (33). It includes three dimensions and 21 items, namely physical domain, emotional domain, and other domain. The Likert 0–5 scale is used to score the patient according to the degree of impact of the disease on the patient. The total score is 0–105 points. The higher scores indicate worse QoL levels. In this study, the Chinese version of the MLHFQ was used for measurement (34). The Cronbach's alpha coefficient of this scale was 0.881, and the Cronbach's alpha coefficient was 0.898 in this study.

### 2.4 Statistical analysis

All statistical analyses were performed using SPSS 27.0 (IBM SPSS Statistics, Armonk, NY, USA). The significance level was *α* = 0.05. Categorical data were expressed as (*n*) and frequencies (%), and continuous data were expressed as mean ± standard deviation. Independent sample *t*-tests and one-way analysis of variance (ANOVA) were used to compare the mean QoL scores among different participant characteristics. Multiple linear regression was used to analyze the influencing factors of QoL. Pearson correlation analyses were used to test the correlation between HL, self-care confidence, and QoL. The SPSS PROCESS macro (model 4) developed by Hayes was used to analyze the mediation effect (35). In the PROCESS macro, we used HL as the predictor variable, self-care confidence as the mediator variable, and QoL as the outcome variable for mediation effect analysis. The bootstrap method was used to test the mediation effect value, and 5,000 random samplings were performed on the original data. When the 95% confidence interval did not contain 0, it indicated that the mediation effect was significant.

## 3 Results

### 3.1 Common method bias test

Since the data of this study were derived from the self-reports of the research participants, there may be common method bias. Therefore, this study used the Harman one-way test to test the common method bias of the original data. Unrotated exploratory factor analysis was performed on all items of HL, self-care confidence, and QoL. The results showed that there were six factors with characteristic roots greater than one, among which the variance explanation rate of the first largest factor was 35.83%, which was lower than the critical standard of 40% (36). Therefore, there was no obvious common method bias in this study.

### 3.2 Sociodemographic and clinical characteristics

Among the 320 HF patients, 178 (55.6%) were male, 233 (72.8%) were aged ≥60 years, 263 (82.2%) were married, 195 (60.9%) were from rural areas, 167 (52.2%) had an education level of primary school or below, 284 (88.75) were unemployed, 185 (57.8%) had an average monthly income of <3,000 CNY, 201 (62.8%) had a LVEF <40%, 173 (54.1%) had a NYHA functional class III, 155 (48.4%) had 1–2 comorbidities, and 125 (39.1%) had a HF duration of 1–5 years, as shown in Table 1.

**Table 1:** Sociodemographic and clinical characteristics.

Variable	*n* (%)	QoL	Statistics	*P*
Gender			*t* = −1.392	0.165
Male	178 (55.60)	47.58 ± 13.44		
Female	142 (44.40)	49.66 ± 13.13		
Age (years)			*F* = 11.95	<0.001
18–44	12 (3.75)	38.08 ± 13.15		
45–59	75 (23.44)	43.77 ± 12.57		
≥60	233 (72.81)	50.56 ± 12.97		
Marital status			*F* = 7.38	<0.001
Unmarried	4 (1.25)	25.75 ± 7.63		
Married	263 (82.19)	47.90 ± 12.71		
Divorced	12 (3.75)	48.33 ± 16.41		
Widowed	41 (12.81)	54.63 ± 13.64		
Living area			*t* = 2.47	0.014
Rural	195 (60.94)	49.96 ± 13.17		
City	125 (39.06)	46.22 ± 13.30		
Education level			*F* = 8.95	<0.001
Primary school and below	167 (52.19)	52.23 ± 13.79		
Junior middle school	72 (22.50)	46.29 ± 11.43		
Senior high school	54 (16.88)	44.35 ± 11.17		
Professional training college	22 (6.88)	38.91 ± 11.71		
Undergraduate and above	5 (1.56)	43.00 ± 11.31		
Work status			*t* = −5.81	<0.001
Working	36 (11.25)	38.31 ± 10.91		
Unemployed	284 (88.75)	49.80 ± 13.06		
Average monthly income (CNY)			*F* = 19.02	<0.001
<3,000	185 (57.81)	52.14 ± 13.73		
3,000–5,000	73 (22.81)	44.81 ± 10.25		
>5,000	62 (19.38)	42.00 ± 11.68		
LVEF			*F* = 4.71	0.010
<40%	201 (62.81)	50.23 ± 13.17		
40%–49%	42 (13.12)	45.12 ± 13.64		
≥50%	77 (24.06)	45.83 ± 12.92		
NYHA functional class			*F* = 29.52	<0.001
II	61 (19.06)	39.77 ± 10.80		
III	173 (54.06)	48.10 ± 12.36		
IV	86 (26.88)	55.50 ± 13.04		
Comorbidities			*F* = 12.38	<0.001
0	23 (7.19)	37.96 ± 11.90		
1–2	155 (48.44)	47.28 ± 12.26		
≥3	142 (44.38)	51.54 ± 13.64		
HF duration (year)			*F* = 7.12	<0.001
<1	120 (37.50)	46.05 ± 12.82		
1–5	125 (39.06)	48.02 ± 12.07		
>5	75 (23.44)	53.24 ± 14.95		

Data are presented as mean ± SD. QoL, quality of life.

### 3.3 The influencing factors of QoL

Multiple linear regression analysis was performed with QoL score as the dependent variable and statistically significant variables as independent variables (age, marital status, living area, education level, work status, average monthly income, LVEF, NYHA functional class, comorbidities, and HF duration). The results showed that average monthly income (*P* = 0.007) and NYHA functional class (*P* < 0.001) were influencing factors of QoL. As shown in Table 2.

**Table 2:** The influencing factors of QoL.

	*B*	*SE*	*β*	*t*	*P*
Constant	16.504	8.232		2.005	0.046
Average monthly income	−3.632	1.333	−0.216	−2.725	0.007
NYHA functional class	5.892	1.141	0.298	5.163	<0.001

B, regression coefficient; SE, standard error; *β*, standardized regression coefficient. The model used the QoL score as the dependent variable.

### 3.4 Scores of HL, self-care confidence and QoL

The HL score was (24.45 ± 9.44) points, the self-care confidence score was (33.66 ± 18.63) points, and the QoL score was (48.50 ± 13.32) points. As shown in Table 3.

**Table 3:** Scores of HL, self-care confidence and QoL.

Variables	Mean ± SD
HL	24.45 ± 9.44
Functional HL	8.55 ± 3.89
Communicative HL	8.77 ± 2.91
Critical HL	7.12 ± 3.29
Self-care confidence	33.66 ± 18.63
QoL	48.50 ± 13.32
Physical domain	23.30 ± 5.94
Emotional domain	8.29 ± 4.80
Other domain	16.91 ± 5.19

HL, health literacy; QoL, quality of life.

### 3.5 The correlation between HL, self-care confidence and QoL

Pearson correlation analysis showed that HL was positively correlated with self-care confidence (*r* = 0.807, *P* < 0.01) and negatively correlated with QoL (*r* = −0.472, *P* < 0.01). Self-care confidence was negatively correlated with QoL (*r* = −0.454, *P* < 0.01). As shown in Table 4.

**Table 4:** The correlation between HL, self-care confidence and QoL.

Variables	HL	Self-care confidence	QoL
HL	1		
Self-care confidence	0.807**	1	
QoL	−0.472**	−0.454**	1

HL, health literacy; QoL, quality of life.

** *P* < 0.01.

### 3.6 Analysis and verification of the mediating effect of self-care confidence between HL and QoL

**Figure 1:**
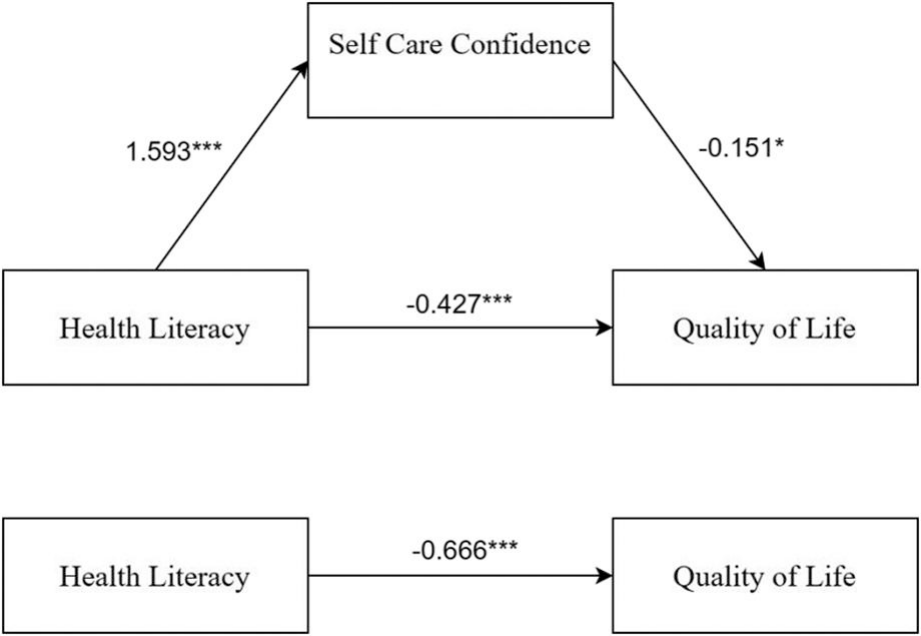
The mediating model diagram of self-care confidence between HL and QoL. *P < 0.05, ***P < 0.001.

As shown in Table 5. HL has a significant negative effect on QoL (*β* = −0.666, *P* < 0.001), confirming our research hypothesis 1. HL has a significant positive effect on self-care confidence (*β* = 1.593, *P* < 0.001). Self-care confidence has a significant negative effect on QoL (*β* = −0.151, *P* < 0.05). When the mediating variable is included, HL also has a significant negative effect on QoL (*β* = −0.427, *P* < 0.001), confirming our research hypothesis 2 that self-care confidence mediates the relationship between HL and QoL. The mediation model diagram for self-care confidence. As shown in Figure 1.

**Table 5:** Analysis of the mediating effect of self-care confidence between HL and QoL.

Variables		Fit index			Significance of regression coefficient		95% CI	
Dependent variable	Independent variable	*R*	*R^2^*	*F*	*β*	*t*	LLCI	ULCI
QoL	HL	0.472	0.223	91.178	−0.666	−9.549***	−0.804	−0.529
Self-care confidence	HL	0.807	0.651	594.451	1.593	24.381***	1.465	1.722
QoL	HL	0.488	0.238	49.584	−0.427	−3.638***	−0.657	−0.196
	Self-care confidence				−0.151	−2.536*	−0.267	−0.034

HL, health literacy; QoL, quality of life; R, correlation coefficient; R^2^, coefficient of determination; *β*, regression coefficient; CI, confidence interval; LLCI, lower level confidence interval; ULCI, upper level confidence interval.

* *P* < 0.05.

*** *P* < 0.001.

The original data were randomly sampled 5,000 times, and the Bootstrap method was used to verify the mediation effect model. The results showed that the total effect value was −0.666, the direct effect value was −0.427, and the indirect effect value was −0.240. The 95% CI of the direct effect value (−0.657, −0.196) did not include 0, indicating that the direct effect was significant, and the direct effect accounted for 64%. The 95% CI of the indirect effect value (−0.446, −0.020) did not include 0, indicating that the indirect effect was significant, and the indirect effect accounted for 36%. This once again proved that our research hypothesis 2 was established, that is, self-care confidence had a mediating effect between HL and QoL. As shown in Table 6.

**Table 6:** Testing the mediating effect of self-care confidence between HL and QoL.

Path	Effect size	SE	95% CI		Effect ratio
			LLCI	ULCI	
Total effect	−0.666	0.070	−0.804	−0.529	
Direct effect	−0.427	0.117	−0.657	−0.196	64%
Indirect effect	−0.240	0.109	−0.446	−0.020	36%

SE, standard error; CI, confidence interval; LLCI, lower level confidence interval; ULCI, upper level confidence interval.

## 4 Discussion

### 4.1 The QoL level of HF patients is low

Our results showed that the QoL score of HF patients was (48.50 ± 13.32) points, which was higher than the results of Zhang et al. (37), indicating that the QoL level of patients in our survey was low (the higher the MLHFQ score, the worse the QoL). This study used multiple linear regression analysis to show that average monthly income and NYHA functional class were important factors affecting patients' QoL. Firstly, in our study, it was found that 57.81% of HF patients had an average monthly income of less than CNY 3,000, which reflects that the income level of most patients was low. In fact, these patients often have insufficient HL levels (14), and the limitation of economic conditions makes these patients unable to obtain sufficient health education and information resources. Furthermore, HF patients need regular medical examinations and continuous drug treatment, which will bring additional financial burden, not only affecting their ability to obtain continuous medical treatment, but also limiting their possibility of seeking better QoL (38). Secondly, in this study, 54.06% of patients were in NYHA functional class III, and 26.88% were in the more serious class IV. The results of the study clearly showed that (39) patients in class III or IV had more severe physical functional limitations and significantly reduced ability to carry out daily activities. Notably, patients with higher NYHA functional class may also have an indirect effect on QoL through depressive symptoms (40). This deterioration in mental state will form a vicious cycle, further increasing the burden of life for patients. Therefore, in order to improve the QoL of HF patients, we can take comprehensive measures to address economic and health challenges, including providing financial assistance, health education (41), and psychological support (42).

### 4.2 HL can significantly affect the QoL of HF patients

As hypothesized in study hypothesis 1, our results showed that HL could significantly predict QoL (*r* = −0.472, *P* < 0.01), which means that the lower the HL level of the patient, the worse the QoL (the higher the MLHFQ score, the worse the QoL). This is consistent with the results of previous studies (43, 44), but different from the results of Zhang et al. (37). One possible explanation is that in the patient population of this study, most patients had a low level of cultural education, and some patients had never received formal education. These patients may face difficulties in reading and understanding medical instructions, such as the instructions for prescription drugs, or encounter obstacles in communicating with medical professionals. Such barriers to communication and understanding may lead to their inability to effectively manage their health conditions, thereby affecting their compliance with treatment and overall QoL (43). Although Zhang et al. (37) concluded that HL was not related to QoL after controlling for covariates in the analysis, their study also pointed out that insufficient HL would lead to reduced QoL. Therefore, improving HL is a key strategy to improve QoL. In the future, medical staff should focus on strengthening health education for HF patients and communication between doctors and patients, which will help improve patients' QoL.

### 4.3 Self-care confidence mediates the relationship between HL and QoL

As hypothesized in study hypothesis 2, our results showed that self-care confidence played a mediating role between HL and QoL, which provided a new perspective for us to understand how HL affects patients' QoL. As a mediating variable, self-care confidence not only reflects the direct effect of HL on QoL, but also reflects its indirect mechanism.

Specifically, on the one hand, having a satisfactory HL level is an important factor in promoting self-care confidence (45). Patients with higher HL levels tend to show stronger self-care confidence (46). This confidence may come from their in-depth understanding of health information and their ability to control health-related decisions. The improvement of HL can enable patients to more effectively obtain, process and apply health information, thereby showing higher initiative and self-care confidence in self-care behaviors (46). On the other hand, the situation-specific theory of HF self-care has proposed that self-care confidence is an important protective factor for self-care behaviors, which is associated with better health outcomes (21). Patients with higher self-care confidence are more likely to actively participate in self-care activities, such as regularly monitoring weight, taking medication as prescribed, and quickly identifying HF symptoms and treating them in a timely manner (25), thereby maintaining a better health state. In addition, patients with higher self-care confidence tend to receive more social support, which includes not only emotional encouragement from family and friends, but also education and resources provided by medical professionals, thereby further enhancing their self-care ability and QoL (19, 24, 47). It is worth noting that the improvement of self-care confidence is also closely related to the patient's psychological well-being. Confident patients are more likely to display a positive attitude and better cope with the stress and challenges associated with HF. This psychological resilience is one of the key factors for successful self-care behaviors (48), which helps patients improve their QoL.

In summary, our findings highlight that improving HL and self-care confidence are key pathways to improving QoL and reducing the risk of adverse clinical events in the daily management of HF patients (49). This finding provides new insights for healthcare professionals in developing and implementing quality-of-life interventions. HL is extremely important for HF patients, and having the corresponding HL can reduce all-cause mortality and hospitalization rates in HF patients to a certain extent, which has positive prognostic significance (50). Therefore, clinical healthcare professionals can incorporate HL improvement into routine HF management processes, and help patients master core skills such as disease knowledge, medication principles, dietary salt restriction, symptom monitoring, and weight management through easy-to-understand individualized health education, simplified instruction manuals, and educational methods combining text, images, and videos, thereby gradually improving their ability to acquire, understand, assess, and apply health information. At the same time, based on literature evidence, it is equally important to improve the HL of caregivers, as caregivers' HL directly and positively affects patients' self-care behavior and QoL (51). Healthcare professionals can include caregivers in the intervention, strengthen their care skills and health information application capabilities through targeted training, build a patient-caregiver collaborative improvement model, and ultimately enhance self-care confidence, improve self-care behavior and improve QoL (51).

Secondly, self-care behavior in chronic diseases is a dual-coping process, and the self-care behaviors of HF patients and their caregivers influence each other (52). Studies have shown that the dual-care model involving caregivers is the core path to enhance patients' self-care confidence (53). According to the dual disease management theory, HF self-management is a dual-process completed collaboratively by patients and caregivers. Medical staff can implement dual-mobile health interventions for patients and their caregivers. Through APP functions, intelligent reminders, personalized feedback and chatbots, continuous interaction can be achieved, enabling patients and caregivers to participate in care plans, share health data in real time, and communicate and make decisions collaborativel (54). This can further optimize the dual-person interaction relationship and strengthen collaborative coping ability, which can significantly enhance patients' self-care confidence, thereby promoting self-care behavior compliance and improving health-related QoL (53). At the same time, this dual-person collaborative care under mobile health support breaks through the limitations of time and space, realizes continuous care and professional-caregiver collaborative support, further consolidates self-care confidence and reduces the risk of adverse clinical event (54).

## 5 Limitations

This study has the following limitations: First, the sample size was relatively small, drawn from only three hospitals in Chengdu using convenience sampling, which may limit the extrapolation of the results. Future studies could explore multi-center, large-sample surveys to improve the broad applicability of the findings. Second, this study relied on patient self-report data. Patients' subjective assessments may be influenced by memory bias and social expectation bias, potentially leading to overestimation or underestimation of their health behaviors, thus introducing errors into the results. Future research could combine objective measurements (such as physiological indicators and electronic medication monitoring records) with self-report data for verification. Third, this study was a cross-sectional design, reflecting only the variable relationships at the time of the survey and unable to infer causal direction. While this study explored the impact and mediating role of HL on self-care confidence, it could not clarify the causal relationship between variables. Future studies could employ longitudinal cohort studies or interventional experimental designs to further verify the causal relationship between variables.

## 6 Conclusion

This study indicates that several demographic variables significantly influence the QoL of patients with HF. Patients with lower average monthly income and higher cardiac function classifications tend to have poorer QoL. This finding suggests that healthcare professionals should prioritize low-income patients and those with poor cardiac function when developing interventions to improve QoL. Secondly, our research enriches the understanding of the relationship between HL, self-care confidence, and QoL, revealing the mediating role of self-care confidence in this relationship. This finding underscores the importance of psychological factors in the self-care process for HF patients. This implies that future care should not only focus on improving the HL of HF patients but also on cultivating their self-care confidence. Providing patient-caregiver-based two-way health education and social support can help patients enhance their self-care confidence and improve their QoL.

## Data Availability

The original contributions presented in the study are included in the article/Supplementary Material, further inquiries can be directed to the corresponding authors.
